# Distress disclosure on social media and depressive symptoms among college students: the roles of social comparison and gender

**DOI:** 10.3389/fpsyg.2025.1520066

**Published:** 2025-04-16

**Authors:** Xiaoli Ye, Haohao Gao

**Affiliations:** ^1^Institute of Higher Education, Anhui University, Hefei, China; ^2^Wuhan Polytechnic, Wuhan, China

**Keywords:** distress disclosure, social media, social comparison, gender, depressive symptoms

## Abstract

**Introduction:**

In contemporary society, individuals are commonly exposed to multiple pressures, under which emotional disorders occur frequently. Especially the upward trend of depressive symptoms among the young population constitutes a non-negligible public health challenge. As social media is increasingly integrated into daily life, individuals’ emotional experiences strongly connect with online interactions. Thus, it is essential to investigate the relationship between the social media usage behavior of young people and their mental health conditions.

**Methods:**

This study conducted an online survey involving 405 college students using the DDI (Distress Disclosure Index), INCOM (Iowa-Netherlands Comparison Orientation Measure), and CES-D (Center for Epidemiologic Studies Depression Scale). It employed a moderated mediation model to explore the connection between distress disclosure on social media and depressive symptoms and the potential roles of social comparison and gender.

**Results:**

The results indicate the following: (1) Distress disclosure on social media is associated with depressive symptoms; (2) Social comparison mediates the relationship between distress disclosure on social media and depressive symptoms among college students; (3) Gender moderates the effect of distress disclosure on social media regarding social comparison, with a more pronounced moderation effect observed in male participants.

**Discussion:**

The findings of this study underscores the importance of adopting appropriate strategies for disclosing distress, fostering healthy tendencies toward social comparison, and recognizing gender differences in mitigating depressive symptoms among young adults.

## 1 Introduction

The rapid acceleration of life pace in contemporary society has profoundly reshaped individuals’ emotional perceptions. This phenomenon has culminated in a compounded effect of multiple stressors experienced ubiquitously by members of society, leading to the frequent emergence of emotional disorders including stress, panic, anxiety, depression, and so on. Approximately 970 million individuals worldwide suffered from mental disorders, with roughly 280 million diagnosed with depression in 2019. Notably, this figure has exhibited a marked upward trend amid the global COVID-19 pandemic (World Health Organization, 2022). Particularly alarming is the shifting demographic landscape of depressive symptoms; a notably younger age group is increasingly affected, posing significant public health challenges that cannot be overlooked. An insightful survey focusing on mental health status among the Chinese population revealed that adolescents are at a significantly higher risk for depression compared to adults, with college students facing particularly pronounced risks (Fu et al., 2023). The urgency and complexity surrounding youth mental health are intricately linked to social media usage (Vidal et al., 2020).

The swift advancement of mobile internet technology and the widespread adoption of smartphones have cultivated a new social landscape. Social media, encompassing both private and public dimensions within cyberspace, serves as a crucial conduit for users to maintain and establish interpersonal relationships while providing a distinctive platform for individuals to express themselves and observe others (Treem et al., 2016). Characterized by low barriers to entry, life-oriented content expression, and concise presentation formats, social media platforms align seamlessly with the fragmented and fast-paced lifestyles prevalent today, becoming deeply integrated into the daily routines of internet users. Currently, there are approximately 5.04 billion social media users worldwide (We Are Social, 2024). Numerous studies have indicated that social media use can alleviate stress, augment sensed social support, and improve subjective well-being (Craig et al., 2021; O’Reilly et al., 2022; Rosen et al., 2022; Sahoo et al., 2024). Nevertheless, contrasting research indicates that excessive or problematic social media engagement exerts adverse impacts on users’ mental health status (Jin et al., 2024; Zhao and Zhou, 2020). A comprehensive meta-analysis further highlights that among young adults, social media engagement is related to increased levels of stress, anxiety, and depression (Shannon et al., 2022). Consequently, while the precise manner in which social media influences mental health remains a subject of persistent scholarly discourse and ongoing investigation, it is undeniable that social media usage significantly shapes an individual’s emotional experiences—both positively and negatively.

In light of this context, exploring the specific behaviors associated with social media usage and their impacts on the young group’s mental well-being is not only an inevitable requirement under the current societal context but also an inescapable and significant topic in the field of mental health research. The current research targets college students to explore the association of specific social media usage behavior, particularly distress disclosure, with their mental health status pertaining to depressive symptoms. Prior studies have established a correlation between distress disclosure and depressive symptoms. Although research on self-disclosure has shifted from offline to online environments following transformations in human interaction patterns, existing investigations remain inadequate in examining distress disclosure within the context of social media. Furthermore, current research exploring the relationship between self-disclosure and depressive symptoms rarely investigates simultaneously the mediating role of social comparison and the moderating effects of gender. Given the prevalent tendencies toward social comparison and gender disparities in online interactions, this study integrates social comparison theory with perspectives on gender differences to elucidate the complex mechanisms underlying the relationship between distress disclosure on social media and depressive symptoms. The objectives are threefold: to promote healthier practices for engaging with social media, to provide empirical evidence for mental health intervention strategies targeting Chinese college students, and to contribute to the expanding body of research concerning social media usage and psychological well-being.

## 2 Literature review and hypothesis development

### 2.1 Distress disclosure on social media and depressive symptoms

Self-disclosure, as a mechanism of information transmission, is intricately interwoven into the fabric of social interactions (Dindia et al., 1997). It encompasses the process through which individuals voluntarily share their inner thoughts, emotional experiences, and personal information with others (Derlega et al., 1993). When such disclosures pertain to personal stress, unhappiness, or distress, they can be classified as distress disclosure (Coates and Winston, 1987), representing a particularly profound form of communication that may possess therapeutic potential. Decades of academic research have continuously shown a significant relationship between self-disclosure and an individual’s physical and mental health (Martin et al., 2020; Aldahadha, 2023; Gonsalves et al., 2023; Doan et al., 2025). For instance, Kahn and Garrison’s (2009) empirical study identified a significant association between emotional self-disclosure and the symptoms connected with depressive symptoms. Similarly, Zhen et al. (2018) discovered that self-disclosure served as a moderating role in the connection between social support and depressive symptoms among flood victims. Specifically, social support primarily alleviates depressive symptoms by enhancing safe sensations and reducing negative self-perception while simultaneously facilitating self-disclosure.

Notably, the domain of self-disclosure has transcended traditional face-to-face interactions and gradually permeated the virtual landscape. With the continuous advancements in internet technology, the construction of individual identities and the formation of relationships have progressively migrated online. Self-disclosure on social media has become an inevitable behavior among contemporary youth within their social interactions. When individuals publicly express emotions, experiences, or states associated with psychological distress on social media platforms (such as Weibo, WeChat, Facebook, etc.) through text, images, or videos, this phenomenon is referred to as distress disclosure on social media. This shift in disclosure contexts has prompted renewed scholarly inquiries into the effects of self-disclosure within online environments. Research indicates that despite changes in communication settings, the impact of self-disclosure on psychological states, including depression, remains significant (Matthes et al., 2021; Sheese et al., 2004). As an example, Piko et al. (2022) signified that Hungarian college students who participated in extensive self-disclosure online were more prone to experiencing depressive symptoms. Michikyan’s (2019) mixed-methods study revealed a correlation between disclosing negative emotions or experiences online and increased depressive symptoms. In view of this evidence, we put forward the subsequent hypothesis:

*H1*: Distress disclosure on social media is significantly related to depressive symptoms.

### 2.2 The mediating role of social comparison

The Social Comparison Theory, formulated by Festinger (1954), advances the proposition that individuals possess an inherent motivation to assess their opinions and abilities by making comparisons with those of others. Schachter (1959) expanded upon this foundational perspective by integrating emotions into the comparative framework, proposing that individuals may participate in comparison to evaluate and comprehend their emotional states when confronted with novel or ambiguous feelings, particularly in the absence of clear physiological or experiential markers for interpretation. As research on social comparison continues to evolve, various dimensions of human life—including academic performance, eating behavior, and appearance—have increasingly been incorporated into the scope of the investigation (Polivy, 2017; Ibn Auf et al., 2023; Lalot and Houston, 2025). To some extent, we can conceptualize social comparison as a psychological activity that is unconsciously triggered within individuals and persists throughout their social interactions.

Drawing upon the advancements in internet technology, the scope of human social interaction has expanded to unprecedented levels. Existing research indicates that social comparison is a prevalent psychological tendency during social media usage (Guo et al., 2022; Kavaklı and Ünal, 2021). For instance, Kirkpatrick and Lee (2024) posited that many mothers participate in social comparisons with the idealized portrayals of motherhood on social media, which can have negative effects on them. Jiotsa et al.’s (2021) research focused on adolescents and young adults determined that social comparison on social media might influence mental well-being by worsening body dissatisfaction and heightening the pursuit of thinness. Concurrently, it has been established that social comparison significantly contributes to depressive symptoms (Wang et al., 2020; Samra et al., 2022; Kagan et al., 2025). A meta-analysis encompassing 14 studies involving clinical populations demonstrates that depressive symptoms are vulnerable to the effects of social comparison (McCarthy and Morina, 2020). Recently, Ruan et al.'s (2023) cross-sectional study examining the mental health of Chinese adolescents revealed that social comparison acts as a mediator in the connection between self-acceptance and depressive symptoms.

Generally speaking, social comparison can be categorized into upward social comparison, downward social comparison, and parallel social comparison. Among these categories, downward social comparisons—where individuals compare themselves to those who perform worse—are typically associated with a positive mental state (Rai et al., 2023). In contrast, upward social comparisons—where individuals compare themselves to those who perform better—are often linked to negative mental health (Tian et al., 2024). It is important to note that individuals in negative emotional states are more prone to engage in upward social comparisons and tend to provide more critical self-evaluations (Swallow and Kuiper, 1988; Burnell et al., 2024). When users express their distress on social media platforms, the unconscious tendency for social comparison may prompt them to ruminate on their distress, thereby exacerbating negative emotions and potentially leading to depressive symptoms (Feinstein et al., 2013; Morina et al., 2024). Thus, the subsequent hypothesis is put forward:

*H2*: Social comparison serves as a mediator in the connection between distress disclosure on social media and depressive symptoms.

### 2.3 The moderating role of gender

Gender differences must be taken into account when conducting emotion-related studies (Kret and De Gelder, 2012). As a crucial element of interpersonal communication, self-disclosure presents a complex and multifaceted array of gender differences that are related to various contexts and methodological approaches (Valkenburg et al., 2010; Liu et al., 2024; Cöbek et al., 2025). The extensive meta-analysis conducted by Dindia and Allen (1992), which examined 205 studies pertinent to self-disclosure, revealed a consistent pattern indicating that females exhibit a higher frequency of self-disclosure behaviors compared to males. In observational studies, females disclosed more information than their male counterparts, regardless of whether the disclosure was directed toward a close acquaintance or a non-intimate individual. Conversely, in studies utilizing self-report methods, female disclosers reported higher levels of disclosure to intimate targets compared to males; however, the level of disclosure was comparable between genders when the target was a stranger. A recent investigation further explores significant gender disparities in patients’ self-disclosure of medical histories through social media platforms against the backdrop of the COVID-19 pandemic in China (Wang et al., 2023), providing new insights into the gender characteristics associated with self-disclosure behaviors within specific contexts.

Additionally, gender disparities significantly influence how people participate in social comparison, thereby modeling both the psychological experiences and behavioral outcomes associated with this process (Thorisdottir et al., 2019; Samra et al., 2022; Sun et al., 2022). Nesi and Prinstein (2015) demonstrate that female users are more inclined to engage in comparisons than their male counterparts when utilizing social networking sites. Research into online body image reveals that, unlike men, women exhibit a greater propensity to edit their photographs and experience heightened negative emotions following instances of upward social comparison (Fox and Vendemia, 2016). A survey examining eating disorder behaviors among junior high school students in China corroborates these findings (Xiang and Kong, 2024). Therefore, given the pervasive nature of gender differences in social interactions, we advance the subsequent hypothesis:

*H3*: Gender serves as a moderator in the relationship between distress disclosure on social media and depressive symptoms.

### 2.4 The current study

**Figure 1:**
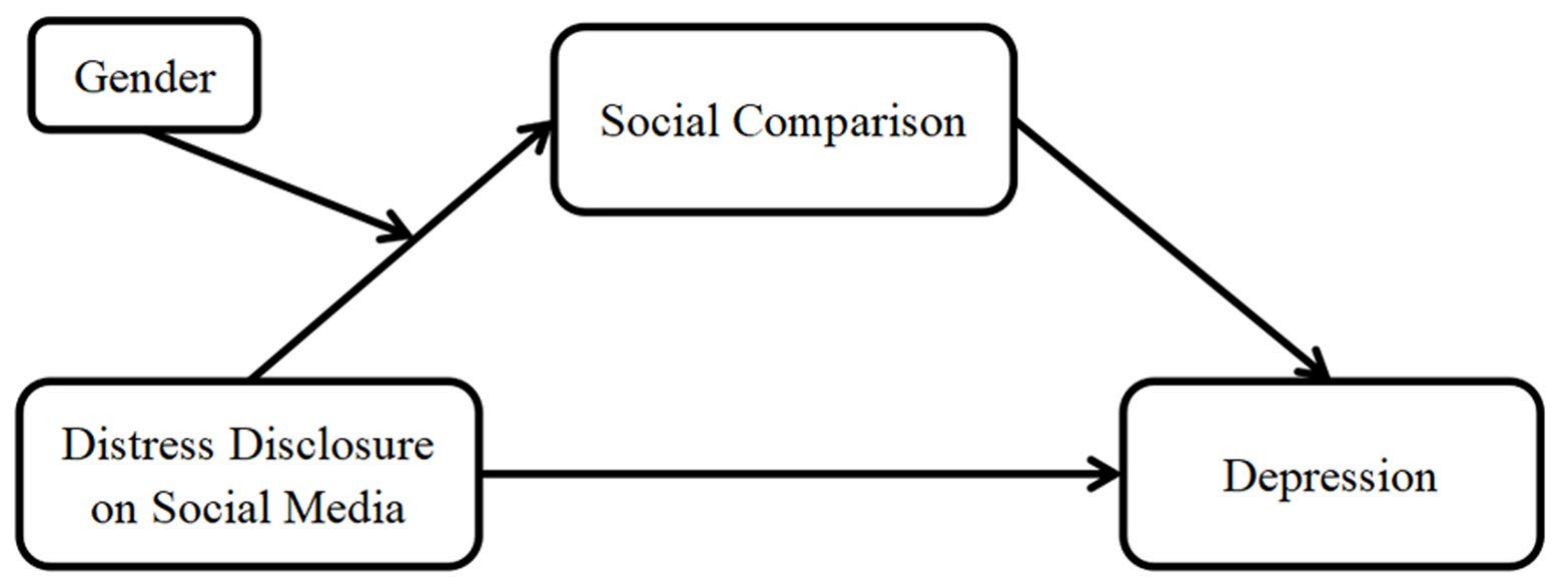
The proposed moderated mediation model.

This research aimed to achieve three primary objectives: (a) to evaluate the impact of college students’ distress disclosure on social media regarding the progression of depressive symptoms; (b) to investigate whether social comparison is a mediating factor in the link between distress disclosure on social media and depressive symptoms among college students; (c) to assess the moderating impact of gender on the connection between distress disclosure on social media and social comparison. A research model was constructed (Figure 1), as asserted in a comprehensive review of the existing literature.

## 3 Methods

### 3.1 Participants

The research was conducted through a questionnaire survey, which included sections on personal information, distress disclosure on social media, tendencies for social comparison, and an evaluation of depressive symptoms experienced over the past week. We collaborated with counselors from several universities in Anhui and Hubei provinces in China from August to September 2024 to distribute the questionnaires among their student populations. The survey was conducted anonymously through the Wenjuanxing online platform, one of the most widely used questionnaire platforms in China. After excluding incomplete or invalid responses (questionnaire completion time less than 60 s), we ultimately gathered a total of 405 credible responses. Table 1 provides a detailed overview of the demographic features of the sample population.

**Table 1:** Sample demographics (*N* = 405).

Category	Number (%)
Gender	
Male	115(28.4%)
Female	290(71.6%)
Education level	
Undergraduate student	309(76.3%)
Postgraduate student	96(23.7%)
Social media usage duration over the past week (per day)	
Less than 2 h	81(20.0%)
2–3 h	98(24.2%)
3–4 h	59(14.6%)
4–5 h	56(13.8%)
More Than 5 h	111(27.4%)

### 3.2 Measurement

#### 3.2.1 Distress disclosure on social media

We conducted the adaptation of the Distress Disclosure Index (DDI) for its Chinese-language version. While retaining the core construct of emotional disclosure, we transition the behavioral context from offline interpersonal interactions to a social media environment. We implement a system scenario conversion strategy that includes: (1) maintaining unchanged emotional trigger conditions (the “when” clause); (2) transforming interpersonal actions (confiding in friends) into platform-specific behaviors (confiding on social media); and (3) ensuring equivalence in mental processes (actively seeking → proactively post). The specific entries of the revised scale are presented in Table 2. The adapted scale contains six items and employs a 5-point for the assessment, with scores spanning from 1 (strong disagreement) to 5 (strong agreement). The outcomes of the confirmatory factor analysis demonstrate robust construct validity for the instrument, as evidenced by the following indices: χ^2^/df = 3.81, CFI = 0.98, TLI = 0.97, RMSEA = 0.08. For the present research, the scale’s reliability was evaluated using Cronbach’s alpha coefficient, which yielded a value of 0.90.

**Table 2:** The distress disclosure index revision.

Original DDI items	Social media adaptation version items
1. When I feel upset, I usually confide in my friends.	1. When I feel upset, I usually confide on social media.
2. When something unpleasant happens to me, I often look for someone to talk to	2. When something unpleasant happens to me, I often talk about these things on social media.
3. I try to find people to talk with about my problems.	3. I try to talk about my problems on social media.
4. When I am in a bad mood, I talk about it with my friends.	4. When I am in a bad mood, I talk about it on social media.
5. I usually seek out someone to talk to when I am in a bad mood.	5. I usually express myself through social media when I am in a bad mood
6. I am willing to tell others my distressing thoughts.	6. I am willing to post my distressing thoughts on social media.

#### 3.2.2 Social comparison

The Iowa-Netherlands Comparison Orientation Measure (INCOM) was utilized to assess the extent of social comparison in college students’ social media usage. The INCOM scale comprises 9 questions that evaluate both abilities (e.g., “I often compare my achievements in life with those of others”) and opinions (e.g., “I often try to figure out what others might be thinking when they face a problem similar to mine”). Responses are scored utilizing a 5-point Likert scale, wherein a rating of 1 signifies “complete disagreement” and a rating of 5 denotes “complete agreement.” Confirmatory factor analysis findings suggest that the scale possesses strong construct validity, as evidenced by the fit indices (χ^2^/df = 2.27, CFI = 0.98, TLI = 0.97, RMSEA = 0.06). For the present investigation, the subscales’ reliability was assessed using Cronbach’s alpha coefficient, which yielded values of 0.86 and 0.84, respectively.

#### 3.2.3 Depressive symptoms

The Center for Epidemiologic Studies Depression Scale (CES-D) was utilized to quantify depressive symptoms among college students over the past week. The CES-D encompasses 20 items, with each item offering four response options that scored from 1 to 4, where a larger numerical value signifies increased frequency. Notably, four of these items are scored in reverse. An increased total score signifies a greater level of depressive symptoms. The results of the confirmatory factor analysis indicate that the scale possesses robust construct validity, characterized by fit indices (χ^2^/df = 3.19, CFI = 0.88, TLI = 0.87, RMESA = 0.07). The Cronbach’s *α* coefficient for this scale was 0.89, providing evidence of the scale’s reliability in the current study.

## 4 Measurement procedures and data analysis

After conducting a confirmatory factor analysis and internal consistency tests on the adopted scales using Amos 28.0, the collected data were subjected to reliability tests, descriptive statistics, and correlation analyses using SPSS 27.0. Additionally, the investigation of specific moderated and mediated effects was conducted using Models 4 and 7 from the SPSS PROCESS macro, as outlined by Hayes (2013).

## 5 Results

### 5.1 Common method bias test

Harman’s one-factor method was employed to evaluate the presence of common method bias. The outcomes of the unrotated exploratory factor analysis revealed six factors, each with initial eigenvalues greater than 1. Among these, the proportion that constitutes the most significant factor accounts for 21.94% of the total variance, falling beneath the 40% threshold level. Consequently, this study does not exhibit substantial common method biases.

### 5.2 Descriptive statistics and correlation analysis

Table 3 offers a presentation of the descriptive statistics and correlation analysis pertaining to the study variables. A statistically significant relationship was observed among the examined variables. In particular, college students’ distress disclosure on social media has a notable positive correlation with social comparison (*r* = 0.29, *p* < 0.01), as well as a noteworthy close relationship between their social comparison and levels of depressive symptoms (*r* = 0.12, *p* < 0.05).

**Table 3:** Descriptive statistics and correlations analysis (*N* = 405).

Variables	*M*	*SD*	1	2	3
1 Distress Disclosure on Social Media	17.66	5.63	-		
2 Social Comparison	29.69	6.41	0.29^**^	-	
3 Depressive symptoms	36.00	9.58	0.09	0.12^*^	-

*p < 0.05; **p < 0.01.

### 5.3 Testing for mediation effect

Model 4 from the SPSS PROCESS macro was employed to examine how social comparison mediates the link between distress disclosure on social media and depressive symptoms in college students while controlling for gender and educational level. Distress disclosure on social media in Model 1 was significantly linked to depressive symptoms (*b* = 0.17, *t* = 2.00, *p* < 0.05), as indicated in Table 4, providing support for Hypothesis 1. The analysis from the model incorporating social comparison as a mediating variable revealed that distress disclosure on social media in Model 2 significantly is related to social comparison (*b* = 0.33, *t* = 6.10, *p* < 0.01). Furthermore, social comparison in Model 3 positively linked to depressive symptoms (*b* = 0.15, *t* = 2.00, *p* < 0.05), whereas the direct pathway of distress disclosure on depressive symptoms became non-significant in Model 3 (*b* = 0.12, *t* = 1.33, *p* > 0.05). The total effects, direct effects, and indirect effects of the mediation model are presented in Table 5. It is observed that the direct effect of distress disclosure on depressive symptoms is not statistically significant [95% CI = (−0.06, 0.29)]; however, both the total effect and the indirect effects are found to be significant [95% CI = (0.00, 0.34), 95% CI = (0.00, 0.11)]. Thus, Hypothesis 2 is supported: social comparison function in the capacity of a mediating variable between distress disclosure on social media and depressive symptoms (indirect effect = 0.05).

**Table 4:** Testing the mediation effect of social comparison on depressive symptoms.

	Depressive symptoms (Model 1)		Social comparison (Model 2)		Depressive symptoms (Model 3)	
	*b*	*t*	*b*	*t*	*b*	*t*
Gender	−2.15	−2.02*	0.53	0.77	−2.23	−2.10*
Education level	−0.50	−0.44	−0.98	−1.33	−0.35	−0.31
Distress disclosure on social media	0.17	2.00*	0.33	6.10**	0.12	1.33
Social comparison					0.15	2.00*
*R* ^2^	0.02		0.09		0.03	
*F*	2.73		12.7		3.07	

*p < 0.05; **p < 0.01.

**Table 5:** The total, direct and indirect effect of distress disclosure on depressive symptoms.

	Coefficient	Boot SE	Bootstrap 95% CI
Direct effect	0.12	0.09	[−0.06, 0.29]
Indirect effect	0.05	0.03	[0.00, 0.11]
Total effect	0.17	0.08	[0.00, 0.34]

### 5.4 Moderated mediation effect analysis

**Figure 2:**
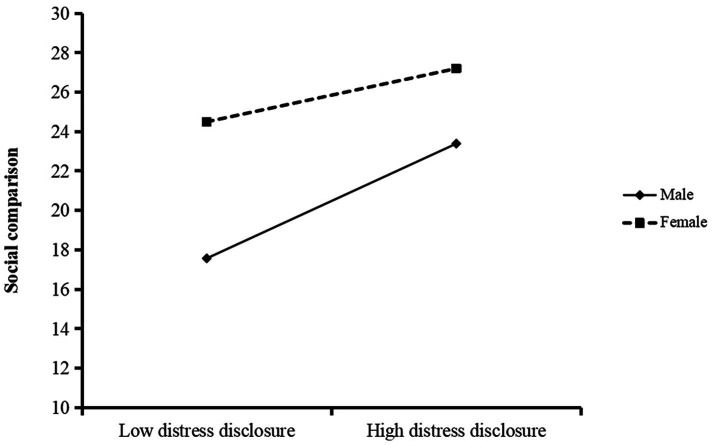
Moderating the role of gender in the relationship between distress disclosure and social comparison.

Hypothesis 3 posited that the impact of distress disclosure on social comparison is linked to gender. To evaluate this moderated mediation model, we utilized Model 7 of the SPSS PROCESS macro to evaluate this moderated mediation model, controlling for education level as a variable. The findings from the gender moderation analysis can be found in Table 6. The interaction between distress disclosure on social media and gender was identified as significant (*b* = −0.28, *t* = −2.42, *p* < 0.05), suggesting that gender indeed moderates the impact of distress disclosure on social comparison. Simple slope analyses revealed a significant association between distress disclosure and social comparison among both males [b_simple_ = 0.52, SE = 0.09, *t* = 5.56, *p* < 0.01, 95% CI (0.33, 0.70)] and females [b_simple_ = 0.24, SE = 0.07, *t* = 3.59, *p* < 0.01, 95% CI (0.11, 0.37)] college students; however, this correlation was notably stronger among males than females (Figure 2). Consequently, Hypothesis 3 is supported.

**Table 6:** Testing the moderated mediation effect.

	Social Comparison		Depressive Symptoms	
	*b*	*t*	*b*	*t*
Education level	−0.99	−1.36	−0.76	−0.67
Distress disclosure on social media	0.52	5.56**	0.12	1.31
Gender	5.37	2.54*		
Distress disclosure on social media ×Gender	−0.28	−2.42*		
Social comparison			0.15	1.91*
*R* ^2^	0.10		0.02	
*F*	11.15		2.60	

*p < 0.05; **p < 0.01.

## 6 Discussion

Through the development of a moderated mediation model, this study investigated how college students’ distress disclosure on social media impacts their depressive symptoms. Specifically, it elucidated the role of social comparison as a mediator and investigated how gender moderates this mediation process.

### 6.1 Distress disclosure on social media and depressive symptoms

The results of our research highlight a notable correlation between distress disclosure on social media and depressive symptoms, particularly when social comparison variables are disregarded. Specifically, students who frequently share negative emotions or experiences on social media demonstrate a markedly increased risk of developing depressive symptoms. This finding sharply contrasts with previous research that primarily examined the relationship between offline self-disclosure and depressive symptoms, which often reported negative associations. However, our conclusions align with previous studies focusing on online environments. These studies suggest that, unlike offline disclosure, self-disclosure and social interactions occurring in internet-mediated contexts may not yield the same benefits (Towner et al., 2022). They may even provoke negative emotions (Chu et al., 2022) and diminish perceived happiness (Schiffrin et al., 2010). Especially for individuals facing adversity, who may be influenced by an array of factors, including false disclosures and limited social support networks, their online distress disclosures are unlikely to enhance their mental health status and may exacerbate feelings of depressive symptoms (Luo and Hancock, 2020).

This may stem from the relatively straightforward nature of the self-disclosure environment in offline, face-to-face communication contexts, which contrasts sharply with the heightened vulnerability of sharing personal information via social media platforms to a myriad of intricate and multifaceted influencing factors. In offline settings, simplicity arises from the physical constraints and direct interpersonal dynamics that shape the disclosure environment. In contrast, social media operates within a broader, dynamic digital landscape, where self-disclosure is intricately associated with a diverse array of individual-level factors (e.g., age, gender, technical skills, emotional state, privacy concerns) and external variables (including disclosure context, anonymity, size of the online network, culture background) (Ashuri and Halperin, 2024), thereby exhibiting a more complex and nuanced interplay of factors. The result reinforces the potential impact of online environments’ characteristics on individuals’ mental health status, particularly in facilitating or exacerbating the transformation of negative emotions into depressive sentiments.

### 6.2 The mediating role of social comparison

Acknowledging the intricacies of the online disclosure environment, this study delves into the underlying mechanisms of social comparison in mediating the connection between college students’ distress disclosure on social media and depressive symptoms. By constructing a theoretical model that incorporates social comparison as a mediating variable, we uncover that when social comparison is incorporated into the model, the existing direct positive correlation between distress disclosure and depressive symptoms is no longer evident, signifying social comparison’s role as a full mediator in this relational chain. In other words, the subsequent elicitation of depressive symptoms among college students after their distress disclosure on social media primarily occurs through an indirect pathway—the process of social comparison. This finding reinforces prior research positing that social comparison behaviors on online platforms constitute a ubiquitous phenomenon, profoundly influencing individual’s emotional experiences, manifesting in intensified jealousy, eroded self-esteem, and the precipitation of depressive sentiments, among other facets (Coyne et al., 2017; Wang et al., 2020; Samra et al., 2022).

On one hand, college students in the emerging adulthood stage experience a life phase characterized by a rich tapestry of diverse choices. The ongoing process of refining and adjusting their visions for future life trajectories not only renders this period exceptionally emotionally intense but also introduces a heightened degree of instability (Arnett, 2004). On the other hand, mobile cyberspace has ushered in an unprecedented era of expressive freedom and discourse empowerment for users, significantly facilitating the circulation and dissemination of information. Nevertheless, this increased engagement and visibility have also given rise to a series of challenges. Driven by the attention economy, exaggerated narratives, the proliferation of one-sided information, and the fabrication of false content have converged to create a complex and dynamic information landscape. Within this intricate context, emotionally nuanced and volatile college students who utilize social media platforms as avenues for personal emotional catharsis or expressions of distress risk becoming ensnared in negative emotions such as depressive symptoms if they do not adequately address the spontaneously arising tendencies toward social comparison. This situation poses a substantial threat to individuals’ physical and mental well-being.

### 6.3 The moderating role of gender

Furthermore, our study found that the interaction between gender and distress disclosure was associated with social comparison. Specifically, within the mediational pathway of “distress disclosure on social media - social comparison - depressive symptoms,” gender plays a moderate role in the initial segment of this pathway. This highlights a pronounced disparity in the relationship between distress disclosure behaviors on social media and social comparison among individuals of different genders. In particular, the correlation is more pronounced among male college students, while it appears to be relatively weaker for female students. In other words, male college students exhibit heightened sensitivity and vulnerability to social comparison when expressing distress on social media platforms compared to their female counterparts. This dynamic may potentially exacerbate their risk of developing depressive symptoms.

This phenomenon may arise from the differing coping mechanisms employed by males and females in managing distressing emotions. Compared to females, males tend to engage less frequently in emotional expression in their daily lives (Buck et al., 1974; Kisilevich et al., 2011), let alone disclose their painful experiences or negative emotions. Within the Chinese cultural context, the traditional notion that “men do not easily shed tears” serves as a microcosm of societal expectations, reinforcing norms that prescribe men to maintain an image of independence, resilience, and bravery. Consequently, this cultural norm propels males towards adopting a more conservative approach to emotional expression, often leading them to avoid disclosing their inner distress to others so that reduce the likelihood of facing harsher societal judgments. In contrast, female self-disclosure tends to elicit greater emotional empathy and networking support than male self-disclosure (Lowenstein-Barkai, 2020). The relatively supportive environment encourages females to share their distress on social media platforms (Tifferet, 2020). This cycle of emotional expression and receipt of positive feedback facilitates effective venting of negative emotions among females, thereby reducing the need for seeking validation through social comparison. In addition, compared to their male counterparts, females exhibit a preference for utilizing a broader spectrum of emotion regulation strategies (Tamres et al., 2002), which encompass but are not limited to rumination (recurring contemplation on emotional events and their implications), positive self-talk (engaging in self-motivation and positive reframing when confronting negative emotions), and cognitive reappraisal (actively adjusting one’s understanding and evaluation of emotional events to diminish their emotional impact). This gender-specific divergence in the adoption of emotion regulation strategies is likely to impact individuals’ patterns of distress disclosure within social media settings, ultimately contributing to gender differences in the extent of social comparison manifested.

### 6.4 Theoretical and practical contributions

This study contributes theoretically in several ways. Firstly, we affirm that individuals’ distress disclosure is related to their depressive symptoms within the increasingly ubiquitous platform of social media, despite potential inconsistencies with findings derived from offline contexts. This revelation underscores the complex and distinctive impact that social media has on the mental well-being of individuals. Furthermore, by innovatively integrating social comparison and gender variables into an analytical model, we separately dissect the mediating or moderating roles they played in the relationship between distress disclosure on social media and college students’ depressive symptoms. This not only deepens our comprehension of the dynamic mental health shifts among college students within social media environments but also offers fresh perspectives and potential explanatory pathways for the observed trend of younger age groups experiencing depressive symptoms, attributed to social media factors. Ultimately, leveraging empirical data gathered from a particular group of Chinese college students, our research enriches the cross-cultural, cross-situational research framework that explores the link between social media usage and mental well-being, contributing valuable empirical insights to the global discourse.

Practically, this research holds significant implications. Specifically, it uncovers that college students’ distress disclosure on social media exacerbates depressive symptoms by intensifying social comparison tendencies, pinpointing crucial intervention targets for mental health. Consequently, we propose several targeted recommendations: Firstly, strengthen mental health education for college students, particularly regarding healthy social media interactions, including guiding them to shift their social comparison mindset and advocating for treating others as sources of learning rather than mere objects of comparison, thereby alleviating the psychological burden stemming from misguided comparisons. Secondly, encourage more positive and constructive strategies during distress disclosure on social media, such as seeking empathy and support rather than mere validation or comparison, to optimize self-cognition and emotion regulation. Additionally, acknowledging the pronounced role of gender differences in the distress disclosure-depressive symptoms nexus, we suggest that mental health service systems should enhance gender sensitivity, offering tailored psychological support and intervention plans for students of different genders to more effectively promote their mental well-being. Lastly, advocate for diversified avenues and methods of seeking help, encouraging students to flexibly choose online or offline channels for emotional expression and problem-solving, thereby comprehensively addressing depressive sentiments and safeguarding mental health.

### 6.5 Limitations and future study

The study has several remaining limitations. Firstly, to mitigate the confusion expressed by participants regarding questionnaire items during the initial survey phase, the DDI and INCOM scales utilized in this research excluded reverse items from the original scale. Consequently, future endeavors should employ the full original scale, or develop a special Distress Disclosure on Social Media Scale to achieve a more comprehensive and objective assessment of participants’ distress disclosure levels. Secondly, being a cross-sectional study, the data collected solely reflects the status of participants at the time of investigation, and the findings cannot be solely interpreted as rigorous causal relationships. Therefore, future research should design more rigorous longitudinal or experimental studies to explore the correlations and underlying mechanisms between variables over an extended period. Lastly, this study focused solely on how social comparison performs a function in the connection between college students’ distress disclosure on social media and depressive symptoms. Given the various factors that influence depressive symptoms within the complex interaction contexts created by social media, the forthcoming investigation should probe additional potential ways in which distress disclosure influences depressive symptoms, offering insights to enhance the mental state of college students.

## 7 Conclusion

Our paper indicates that the distress disclosure on social media platforms among college students significantly is related to their depressive symptoms, with this mediation role played by the psychological tendency toward social comparison. Furthermore, this research confirms that gender act as a moderated role in the connection between distress disclosure on social media and depressive symptoms, suggesting that male students’ disclosures have a more pronounced impact on their tendencies for social comparison compared to their female counterparts. Consequently, universities and families should pay close attention to students’ use of social media, enhancing their media literacy by guiding them towards healthier practices and accurate discernment of online content. This approach is likely to foster positive effects related to social comparison, ultimately mitigating depressive symptoms and bolstering self-esteem.

## Data Availability

The raw data supporting the conclusions of this article will be made available by the authors, without undue reservation.
